# Disentangling effects of abiotic factors and biotic interactions on cross-taxon congruence in species turnover patterns of plants, moths and beetles

**DOI:** 10.1038/srep23511

**Published:** 2016-04-01

**Authors:** Meichun Duan, Yunhui Liu, Zhenrong Yu, Jacques Baudry, Liangtao Li, Changliu Wang, Jan C. Axmacher

**Affiliations:** 1 College of Agricultural Resources and Environmental Sciences, China Agricultural University, Beijing, China; 2 SAD Paysage, French National Institute of Agronomic Research (INRA), Rennes, France; 3 College of Agriculture, Hebei Unversity of Engineering, Handan, China; 4 Department of Architecture and Urban Planning, Southwest University for Nationalities, Chengdu, China; 5 Department of Geography, University College London, London, UK

## Abstract

High cross-taxon congruence in species diversity patterns is essential for the use of surrogate taxa in biodiversity conservation, but presence and strength of congruence in species turnover patterns, and the relative contributions of abiotic environmental factors and biotic interaction towards this congruence, remain poorly understood. In our study, we used variation partitioning in multiple regressions to quantify cross-taxon congruence in community dissimilarities of vascular plants, geometrid and arciinid moths and carabid beetles, subsequently investigating their respective underpinning by abiotic factors and biotic interactions. Significant cross-taxon congruence observed across all taxon pairs was linked to their similar responses towards elevation change. Changes in the vegetation composition were closely linked to carabid turnover, with vegetation structure and associated microclimatic conditions proposed causes of this link. In contrast, moth assemblages appeared to be dominated by generalist species whose turnover was weakly associated with vegetation changes. Overall, abiotic factors exerted a stronger influence on cross-taxon congruence across our study sites than biotic interactions. The weak congruence in turnover observed particularly between plants and moths highlights the importance of multi-taxon approaches based on groupings of taxa with similar turnovers, rather than the use of single surrogate taxa or environmental proxies, in biodiversity assessments.

Obtaining a good understanding of the mechanisms that underlie species richness and species composition presents one of the most significant challenges facing ecologists, biogeographers and conservation planners alike^1^,^2^. This challenge is linked to the fundamental question whether species richness and changes in species assemblages are spatially congruent across taxonomic groups^3^,^4^. High congruence levels in the spatial patterns of either species richness (α-diversity) or species turnover and community similarity (β-diveristy) across taxonomic groups^4^,^8^,^9^ would allow the identification and use of surrogate taxa as taxonomic groups whose diversity patterns are indicative of multi-taxa diversity changes^5^,^6^,^7^. Their identification could greatly aid biodiversity monitoring and conservation planning^10^,^11^, and allow for the identification of general mechanisms underlying spatial variations in biodiversity^12^.

Previous studies of diversity congruence have primarily focused on α-diversity within natural or semi-natural ecosystem, while few studies have focused on the effects of anthropogenic disturbance gradient on cross-taxa congruence in species turnover patterns^12^,^13^,^14^. These studies yielded both taxonomically and geographically highly variable results^13^,^15^, putting into question the actual existence of cross-taxon α-diversity congruence itself, and the validity of associated taxon surrogacy approaches in biodiversity conservation^8^,^16^. Furthermore, α-diversity on its own contains limited biological information^6^,^13^, since assemblages identical in their α-diversity can have a completely different species composition, fulfil very different ecosystem functions and provide distinctly different ecosystem services. Accordingly, the congruence in turnover patterns or community similarity across taxa that can provide important insights for biodiversity conservation^11^,^17^ has recently received increasing attention^10^,^18^,^19^. Respective studies have confirmed an apparent wide-spread, strong congruence in spatial community dissimilarity patterns across taxonomic groups^6^,^13^,^20^, but the underlying causes for these patterns are still poorly understood^17^,^21^,^22^.

High cross-taxon congruence has been related both to similarities in the response patterns of these taxa to changes in environmental variables and to biotic interactions^5^,^19^,^23^,^24^,^25^. However, the relative contributions of environmental abiotic drivers and biotic interactions towards cross-taxon congruence remain widely unknown^21^,^26^. Some studies indicated significant biotic interactions between plant and animal assemblages that persisted even when the effects of environmental factors were controlled^22^,^27^. Thus surrogate taxa could be identified based on the direct biotic interactions such as trophic relationships between target and surrogate taxa^3^,^5^,^23^. Other investigations suggested environmental variables to exert a predominant influence on links between taxon pairs^24^,^28^,^29^. In this context, environmental parameters could be effectively used and potentially also be directly managed in the context of biodiversity conservation^9^,^13^,^21^. The picture becomes further complicated once geographic distance is added as potential explanatory parameter. Geographic distance is commonly regarded as relating to mid- to large-scale environmental gradients^22^,^30^,^31^, and associated with dispersal limitations^26^,^32^. Since these also strongly affect turnover in different taxa with implications for cross-taxon congruence^6^,^30^, neglecting the spatial setting of plots in the analyses of species’ turnover patterns could create misleading results^33^. Few studies have nonetheless tried to disentangle the relative contributions of environmental and biotic factors on cross-taxon congruence while simultaneously controlling for the effects directly related to geographic distance^22^,^33^,^34^.

Our study builds on earlier investigations showing that the α-diversity patterns of four taxonomic groups, vascular plants and three insect taxa (Lepidoptera: Geometridae and Erebidae, subfamily Arctiinae, and Coleoptera: Carabidae), are highly non-congruent in our study area composed of plots situated along both altitudinal and anthropogenic gradients in an anthropogenically transformed mountainous landscape matrix^8^. Here, we now investigate the links and drivers of turnover patterns across these four taxa. Vascular plants as the main group of producers in terrestrial ecosystems are often hypothesized to strongly influence the species composition of consumers across trophic levels via bottom-up effects^5^,^35^. The two moth taxa represent herbivores that are characterized by a strong dispersal ability^36^, while carabids represent a chiefly predatory insect taxon with some granivorous and omnivorous species^37^ that contains a number of flightless species and generally shows a more limited dispersal in comparison to the moths.

In establishing the degree of cross-taxon congruence in species turnover patterns across these four taxonomic groups, we particularly focused on the partial contributions of environmental factors and biotic interactions. We first of all hypothesize that a strong congruency exists between vascular plants and carabid beetles. Both taxa are chiefly composed of weak dispersers, with plants strongly relying on passive dispersal and with most carabid species known to chiefly disperse over limited distances of only up to 50 m^38^. In comparison to highly mobile moths, plants and beetles will therefore be more strongly influenced by local environmental conditions and anthropogenic disturbances. Moreover, we hypothesize that strong congruency also exists between vascular plants and the two moth taxa even the role of environmental factors is controlled for due to the direct trophic link between the plants and herbivorous pollinators. We furthermore aim to identify potential surrogate taxa or envrionmental proxies that allow predictions of turnover patterns across our different taxa.

## Results

### Species composition

We recorded a total of 415 vascular plant species, while 14692 specimens representing 110 species of geometrids and 1543 individuals representing 20 arctiinid species were caught in light traps, and 3663 carabids representing 73 species were sampled in the pitfall traps. Each study site and habitat had its own, unique species of vascular plants. For carabids, all study sites, but not all habitat types at all sites contained unique species. For geometrids, only woodland and grassland habitats harbored unique species. Two sites, Dayushu and Gaojiaying, and none of the habitats harbored unique arctiinid species (Supplementary Table S1).

### Species turnover between study sites and habitats

**Figure 1:**
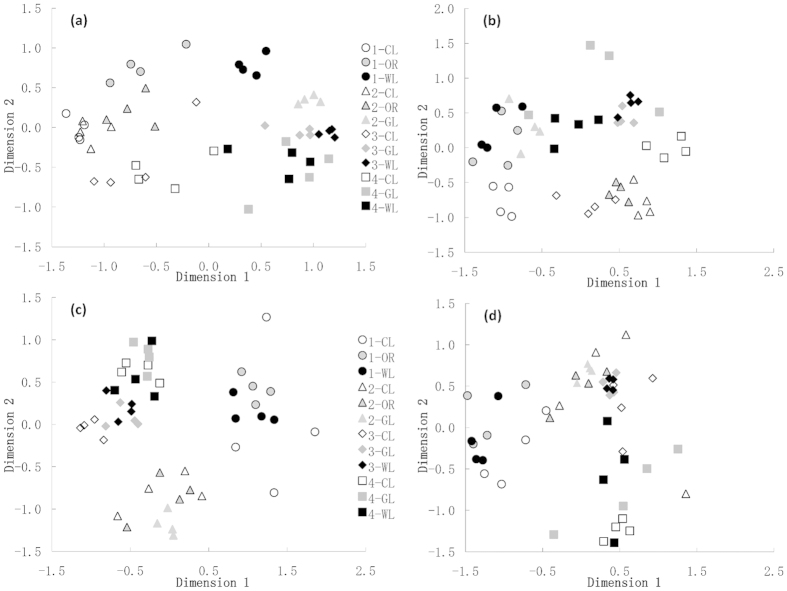
NMDS ordination plots showing the assemblage structure of (**a**) vascular plants (Sørensen dissimilarity, stress: 0.14), (**b**) Carabidae (CNESS *m* = 3, stress: 0.20), (**c**) Geometridae (CNESS *m* = 16, stress: 0.18) and (**d**) Arctiinae (CNESS *m* = 3, stress: 0.19), study sites: circle - Dayushu (1), triangle - Gaojiaying (2); diamond - Baiqi (3); square - Shizigou (4), habitat types: white - cultivated land (CL), close grey with black line - orchard (OR), grey - grassland (GL), black - woodland (WL).

The non-metric multidimensional scaling (NMDS) showed that both moth assemblages formed very distinct study site-specific clusters (Fig. 1c,d). A clear trend for the formation of such clusters was also visible in ground beetles (Fig. 1b) and the vegetation (Fig. 1a), but particularly in the plant communities, there was a higher degree of overlap between plots from different sites. Plant and carabid assemblages recorded on cultivated land also showed a tendency to separate from assemblages recorded at the other habitats across sites, with carabid assemblages on orchards clustering together with the “cultivated land” assemblages. No habitat-specific clusters were observed for either moth taxon (Fig. 1c,d).

### Individual environmental variables as predictors of species turnover

The turnover in all taxonomic groups was significantly correlated with elevation and also the habitat type ‘cultivated land’, with the latter being particularly significant for vascular plants and carabids (P < 0.001). Additionally, vascular plants were significantly correlated with all environmental variables except soil texture and landscape diversity (SHDI). Apart from elevation and cultivated land, carabid turnover was also significantly linked to the % of semi-natural land area (SNP), while arctiinid turnover was additionally correlated with changes in total N and soil texture (Table 1).

### Cross-taxon congruence

All comparisons of turnover patterns between pairs of taxa showed significant positive correlations in 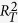 (P < 0.001, Table 2). Species turnover in vascular plants was most strongly linked to carabid turnover, while turnover in geometrids and arctiinids were more closely linked than turnover of either moth taxon with the assemblage changes in carabids or plants. For the congruence correlated with the remaining environmental distances (CTE), reflecting the independent effect of changes in environmental factors on cross-taxon congruence, the model between plants and carabids explained the highest degree of variance (13% and 8%), while the other models explained distinctly lower proportions (<3% of variance). Plant and carabid turnover was also well predicted by PT (the congruence not associated with geographic or environmental distances and hence related purely to proxies of potential biotic interactions), indicating proxies for potential biotic interactions between them, while the PT-link between vascular plants and the two moth assemblages was not significant.

## Discussion

There are two contrasting views on the role of environmental conditions on cross-taxon congruence. A range of studies links significant cross-taxon congruence to common response patterns of the different taxa to changes in key environmental factors^23^, such as available energy^24^, humidity^28^ or grazing regimes^34^. In contrast, other authors argue that different taxa will show highly independent specific responses to changes in environmental conditions^16^,^18^,^19^,^39^, which in turn is often linked to weak congruence^10^,^17^,^20^. The variations in the independent influence environmental factors exert on cross-taxon congruence of different taxon pairs in our study can be related to this divergence in viewpoints and past observations.

The most important environmental determinant of turnover across taxa was elevation, which commonly represents a whole array of environmental factors. These include temperature, radiation and barometric pressure, which all show monotonic responses to changes in elevation, as well as precipitation^40^, where links can nonetheless be much more complex and influenced for example through variations in exposition or the vegetation structure^41^. These environmental changes have implications also for soil parameters, with pH and soil organic matter (SOM) in our study area distinctly changing in relation to changes in elevation. Given this wealth of parameters associated to elevation, it is unsurprising that this factor exerts a strong influence on the turnover of all four target taxa in our study area, which results in the significant explanatory value of elevation in explaining and underpinning inter-taxon congruencies.

Beyond the influence of elevation, the strong overall congruence observed between the turnover in vascular plants and carabids can be explained by both of them additionally reacting strongly towards a similar set of additional environmental variables, particularly to land cultivation and the proportion of semi-natural land present in the plot vicinity. These parameters chiefly reflect the anthropogenic disturbance gradient, with the results confirming our expectations. Cultivation impacted on the species composition of both vascular plants and carabids due to its direct associations with habitat management^42^, including the application of agro-chemicals that are known to exert a strong controlling effect on both these taxa^39^. The strong association between species compositions of plants and carabids with neighboring semi-natural habitats can be linked to the low mobility of the two taxa^43^,^44^. This results in neighboring habitats becoming the main source for species’ colonization. In contrast to plants and carabids, the turnover in the two moth assemblages showed little associations with habitat types and landscape patterns in the vicinity, which we believe relates not only to their greater mobility, but also to the presence of moth assemblages chiefly composed of habitat generalists across the investigated landscape, since other studies have shown a very strong influence of habitat type even across plots in close proximity for geometrid moths^45^. It also appears that with the exception of elevation, plant and moth assemblages react distinctly different to the measured environmental parameters.

Biotic interactions, including trophic interactions, host–parasitoid interactions, but also intraspecific interactions at the same trophic level, may promote cross-taxon congruence^1^,^21^. Observed positive relationships between plants and their consumers are often linked to the ‘food-plant diversity hypothesis’^27^. Here, a positive feedback between plant and herbivore species richness is attributed to an increase in food variety available to the herbivore community. Similar positive feedback links are also hypothesized to connect herbivore assemblages to assemblages at higher trophic levels^1^,^46^. Additionally, increased plant species richness according to the ‘vegetation structure hypothesis’^24^,^25^ also results in an increased complexity of the vegetation structure which offers more structural niches for a wider range of animal species to fill. Theoretical deliberations suggest that changes in plant species communities are associated with changes in both, available food selection for herbivores and structural niches, and should therefore also trigger shifts in the assemblage structure at higher trophic levels. Such strong congruence in compositional changes related to producer-consumer links has already been reported in a number of studies^1^,^6^,^33^.

In contrast to our second hypothesis and these studies, the assemblage structure of the two herbivorous moth taxa which rely heavily on the vegetation as key food resource for their caterpillars and as nectar source of adult moths^47^, shows only weak direct links with the vegetation composition in our study. This is particularly true once the role of abiotic environmental factors has been accounted for. Similar patterns were already observed for the turnover in geometrid moth assemblages on Mt Kilimanjaro^8^ and Changbai Mountain^29^, which primarily relates to abiotic factors such as precipitation and temperature, rather than to direct biotic interactions between the moth and plant assemblages. In our study, the weak direct biotic links between the turnover of plant and herbivore taxa can be explained partly by the widespread loss and degradation of pristine habitats during the agricultural transformations and the historical widespread logging occurring in this region^48^. The associated loss of pristine habitat specialists will have led to the current moth assemblages being mainly composed of generalist species with weaker associations to specific plant species^5^,^29^,^49^. This also supports the general lack of moth habitat specialists mentioned above (Supplementary Table S1). The relatively low species richness in both, the recorded arctiinid, but partly also the geometrid assemblages in comparison to other studies^28^,^36^,^50^, can be seen as indicative of this pattern, too.

With most ground beetles being predatory or omnivorous^37^, carabids and vascular plants widely lack direct food-related relationships. The strong direct links observed between plant and carabid turnover are therefore likely to be related to trends underlying the “vegetation structure hypothesis”^24^,^25^. Previous studies have reported on the importance of the vegetation in determining the physical structure of beetle habitats, providing a variety of microhabitats, shelter, as well as ovipositioning sites^42^,^44^,^51^. These effects will have been integrated in the measured proxies for potential biotic interactions (PT value). Qian *et al.*^31^ suggest that this fraction also reflects common responses of two taxonomic groups to unmeasured environmental factors. Our analyses already included a very wide range of environmental factors via the inclusion of elevation, soil parameters, habitat types and landscape structure. In line with Jones *et al.*^30^ and Özkan *et al.*^21^, we thus believe that our results, suggest the presence of direct biotic links between plants and ground beetles that also support the significant congruence in their turnover patterns.

Our results are indicative of a highly complex and variable interplay of environmental factors and potential biotic interaction in explaining cross-taxon congruence in turnover patterns of the investigated taxon pairs. These differentiations are also expected in relation to spatial scales and biogeographic processes acting within them^2^,^4^. The overall spatial extent and resolution of any investigation is known to strongly affect the relative roles of these mechanisms in explaining congruence in turnover patterns^10^,^12^. For localized studies, biotic interaction are commonly assumed to play a particularly strong role in determining biodiversity patterns, whereas environmental variables are expected to become more important once study areas cover large regions and grain sizes^2^,^5^,^22^. Our study covers a substantial region, where the influences of abiotic environmental variables on the cross-taxon congruence between all taxon pairs with the exemption of the two moth taxa are stronger than influences of the proxies for potential biotic interactions. The crucial role of elevation as explanatory environmental factor for species turnover and the resulting congruence in cross-taxon turnover patterns is furthermore an environmental parameter that is strongly linked to intermediate or large study areas. It can hence be speculated that once this dominant factor is kept relatively constant as expected in small study areas, biotic interactions will indeed show a strongly increased explanatory value for potential congruence in the turnover of our taxonomic groups.

Spatial differentiations in species assemblages also strongly relate to dispersal limitations, with the species composition between two sites generally increasing in dissimilarity with increased geographic distance^26^,^32^. Differences in dispersal strategies and processes in different taxa will therefore further underpin the degree of cross-taxon congruence in species turnover pattern^13^,^30^. Our results only provide some indications of these trends via the distinct pairing of moth assemblages and carabid with vascular plant assemblages, reflecting pairs of strong versus weak dispersers. Future studies could examine the role of dispersal limitations on cross-taxon congruence in species turnover in a spatially explicit manner across different spatial scales. This means that both, the specific ecological traits of the investigated taxa themselves and the spatial dimensions of the investigation, will determine the relative importance different drivers exert on cross-taxon turnover congruence^4^,^52^.

A key finding of our study is the low congruence between vascular plants and herbivorous, pollinating moth taxa in this anthropogenically transformed landscape matrix, especially once the influence of abiotic factors is controlled for. Previous studies across a variety of taxonomic groups, natural ecosystems and spatial scales, have also reported low cross-taxon congruence of species turnover patterns between insect and other taxa or between different groups of insects^4^,^9^,^17^,^20^,^21^,^52^. Thus, the widespread use of surrogate taxa like vascular plants or vertebrates without any further consideration of insects that represent the most species-rich taxa on our planets that also fulfil a plethora of crucial ecological functions must be seen extremely critical. Supplementing earlier reports on the lack of congruence in α-diversity patterns^8^ and general meta-analyses of species richness patterns^4^,^5^,^15^, turnover congruence across taxon-pairs was highly variable and relatively weak, particularly once elevation change as dominant determinant was controlled for. Thus, the potential for ‘single taxon surrogacy’ is clearly limited. Even the use of environmental proxies that suggested by many studies^9^,^13^,^24^,^28^, in this case namely elevation change as environmental biodiversity proxy, is limited. Correlation coefficients between changes in species assemblages and elevation ranged from 0.26–0.35, with much smaller coefficients reported for all other environmental drivers like habitat type. These values are substantially lower than 0.7 that has been proposed as threshold to judge an optimal surrogate^17^,^20^.

However, our results also indicate that the subset of taxa could be representative of biodiversity^4^,^17^. Vascular plants could be used to represent the turnover in carabids, with geometrid and arctiinid moths forming another taxon pair with very similar turnover patterns and responses to environmental and biotic changes. Thus, a combined use of either one of the taxon pairs vascular plants/carabids and geometrids/arctiinids would provide strong implications for the turnover of all four taxa investigated (Supplementary Table S2). The selection of suitable surrogate pairings or groupings will nonetheless require strong insights into the highly differentiated responses of the respective taxa to environmental and biotic changes that occur across different spatial scales, and α- and β-diversity patterns likely require independent consideration. While our study provides some first strong insights in this respect, more research is clearly needed to allow a more meaningful analysis for example of the global hotspots for multi-taxon biodiversity and the main drivers of cross-taxon α- and β-diversity patterns.

## Methods

### Study sites and plot selection

Our study was conducted at four distinct sites in our large study region (40°23′–41°12′ N, 114° 57′–115°57′ E) located within the mountain ranges between Beijing and the Inner Mongolian Plateau in northern China. The four study sites were located within the vicinity of four villages (Dayushu, Gaojiaying, Baiqi and Shizigou) situated at elevations of about 500 m, 900 m, 1400 m and 1650 m, and therefore covering substantial proportions of the regional elevation gradient. Three representative habitat types were subsequently selected at each site according to the prevailing management intensity, natural vegetation and farming regime (Supplementary Table S1 and Axmacher *et al.*^8^ for further details). Studied habitats included cultivated land, regularly plowed and cropped with maize, oat, potato or vegetables and regularly treated with pesticides and chemical fertilizers, fruit orchards intercropped with a variety of other crops and again commonly treated with pesticides and fertilizers, grassland where occasional intrusions from livestock occurred but where grazing was officially prohibited, and woodland that remained widely undisturbed apart from low-level collections of mushrooms and wild vegetables. These habitat types covered the main regional habitat types and anthropogenic disturbance gradients. At each selected habitat type, 4 plots measuring 20 m × 20 m were established, resulting in a total of 12 plots per study site.

### Recording of plants and insects

We recorded the % cover of all vascular plant species in June and September 2007. Trees and shrubs were recorded on the entire plots, while herbaceous species were recorded at five 2 m × 2 m subplots located in the center and four corners of each plot.

Automatic light traps were used for moth sampling between May and October in both 2006 and 2007. These traps consisted of a 12 V-battery operated UV light tube (Sylvania black light-blue, F 15W/BLB-TB; Osram, Munich) placed above a plastic funnel leading into a plastic bucket. The weak light source allows targeted attraction of moths from the direct vicinity of the trap^53^. Moths on each plot were sampled twice per month during 6-day periods around the new moon from 19:00 to 23:00 to avoid the effect of strong moonlight. Ground beetles were sampled using pitfall traps over 6 day-periods every month between May and October in both 2006 and 2007. Eight pitfall traps were placed at a distance of 4 m and 7 m from the plot center along N–S and E–W facing diagonal lines intersecting in the centre of the plot. Pitfall traps were formed of cups 8 cm in diameter and 11.5 cm in depth that were partly filled with 75% alcohol to kill and preserve the specimens. Each trap was protected from rain by an aluminum roof positioned about 5 cm above the trap (see Axmacher *et al.*^8^ for further details).

### Recording of geographic coordinates and environmental parameters

The exact latitude, longitude and elevation of each plot were established using maps and a Garmin GPS III. Differences in latitude and longitude between plots were subsequently transformed into geographic distances and treated separately, while changes in elevation were jointly considered with the other environmental parameters in the analysis. Five soil samples were randomly taken from the upper 20 cm of the mineral soil at each plot and then mixed. These samples were analyzed for their contents in SOM, total nitrogen, and soil pH (see Axmacher *et al.*^8^ for details). The soil texture was classified on a 1–5 scale reflecting increasing soil clay contents. The landscape was characterized using land-use mapping with differentiation of five cultivated land habitat types (maize, oat, vegetable and potato fields as well as orchards), three semi-natural habitats (woodland, bushland and grassland), and other habitats (including roads and buildings), whose extent was recorded in 100 m × 100 m quadrats centered on the individual plots. Fragstats 3.3^54^ was then used to calculate landscape metrics including the proportion of semi-natural land, the proportion of cultivated land and the Shannon-Wiener landscape diversity index based on these habitat types to characterize landscape heterogeneity.

### Data analysis

Dissimilarities of species composition between sampling plots for each of the three insect taxa were calculated based on the Chord-Normalized Expected Species Shared (Gallagher’s CNESS) index. This index is particularly tailored for cases where samples are incomplete, contain many rare species and are of different size and diversity^50^. The CNESS index can be adjusted to varying sample sizes by changing the sample-size parameter *m*. For the respective maximum common sample size (size of the smallest sample) which we used here, the index provides an overview of changes in the structure of the entire assemblage. To investigate changes in vascular plant assemblages where complete sampling can be assumed, a Sørensen dissimilarity matrix based on presence–absence data of all plant species per plot was calculated. All dissimilarity matrices were computed using COMPAH96^55^, and we subsequently visualized the species composition differences between sites and habitats using NMDS computed in Statistica 6.0^56^. Euclidean distances were calculated to generate matrices representing geographic distances and changes in environmental parameters between plots. With the exception of pH, all environmental variables were log-transformed and then standardized using a z-transformation prior to the calculation of the Euclidean distances.

We tested the correlation between the dissimilarity matrices for each taxon and each environmental distance matrices using partial Mantel tests. When significant spatial correlationsin species turnover patterns were found across all four taxa (Supplementary Fig. S1), the geographic distance matrix was employed to control for the intrinsic spatial components of the community structure in the analysis. Spearman rank correlation formed the basis of the Mantel tests since assumptions for parametric tests were not always fulfilled. Since elevation, soil pH and SOM were strongly correlated (|r| > 0.7) (Supplementary Table S3), we only used elevation in our analysis to represent these three parameters, hence avoiding distortions caused by multi-collinearity^57^. Similarly, we selected proportion of semi-natural land from the highly negatively correlated pair of it and the proportion of cultivated land.

**Figure 2:**
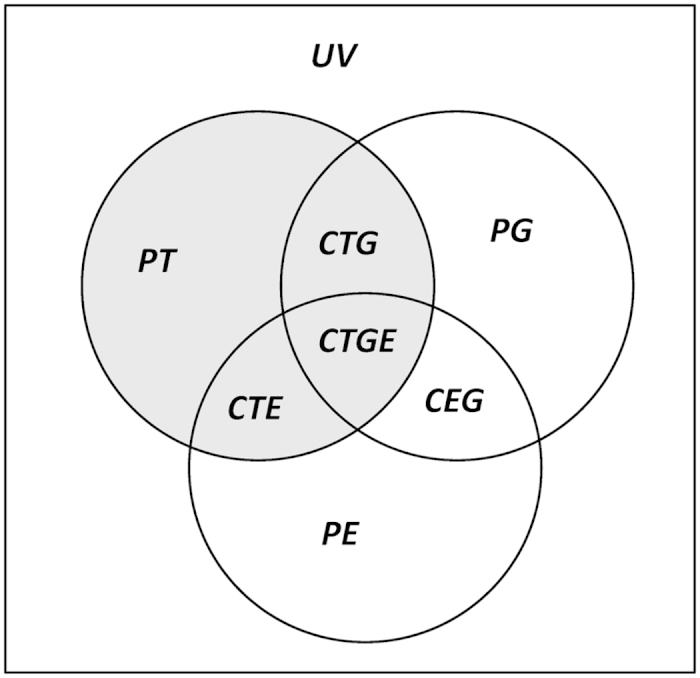
Sketch of the variation partitioning for the variation of taxonomical dissimilarity matrices group into 8 fractions. UV is the unexplained variance. PE, PG and PT are pure variation explained by environmental distances (E), geographic distance (G) and another taxonomical dissimilarity matrix (T), respectively, while CTE, CTG, CEG and CTGE, are fractions indicating their combined effects. The grey circle represents the quantitative congruence between two taxa (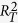).

We finally used variation partitioning in a series of Multiple Regressions on distance matrices (MRMs) to quantify the proportion of variation in taxonomical dissimilarity between plots that can be explained by the dissimilarity matrix of another taxon, as well as by geographic and environmental distance^58^. The variation in taxonomical dissimilarities between pairs of plots was partitioned into eight fractions, based on unique or combined variations explained by these three matrices: (1) PT, pure variation explained by another taxonomical dissimilarity matrix, (2) PE, pure variation explained by environmental distances, (3) PG, pure variation explained by geographic distances, (4) CTE, combined variation explained by another taxonomical dissimilarity and environmental distances, (5) CTG, combined variation explained by another taxonomical dissimilarity and geographic distances, (6) CEG, combined variation explained by environmental and geographic distances, (7) CTGE, combined variation explained by all three matrices and (8) UV, unexplained variance (Fig. 2).

We therefore adopted the procedure introduced by Jones *et al.*^58^, with 7 MRMs computed for each taxon as dependent variable, and unique or combined contributions of cross-taxon matrix correlations with another taxon, geographic and environmental distances as independent variable(s) (Supplementary Table S4). In each respective analysis between pairings of the four taxa, the environmental distance matrix used was calculated by combining all environmental variables that yielded a significant independent matrix correlation with both taxa investigated in the Mantel test, and standardized each environmental variable to give each variable equal weight. The 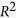-values of each regressions analysis gave an indication of the explained variance by each fraction. As we focused on the cross-taxon congruence between pairs of taxa, only 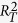 as the quantitative congruence between two taxa was presented. This was formed by the three sections CTG + CTGE (congruence correlated with geographic distance), CTE (congruence correlated with remaining environmental distances) and PT (congruence not associated with geographic and environmental distances and hence related purely to proxies for potential biotic interactions).

All mantel tests and MRMs were calculated using the Ecodist-Package^59^ in R 3.0.2^60^. We used Monte Carlo permutation tests with 1000 runs to establish the significance of Mantel test and MRM results.

## Additional Information

**How to cite this article**: Duan, M. *et al.* Disentangling effects of abiotic factors and biotic interactions on cross-taxon congruence in species turnover patterns of plants, moths and beetles. *Sci. Rep.* **6**, 23511; doi: 10.1038/srep23511 (2016).

## Supplementary Material

Supplementary Information

## Figures and Tables

**Table 1:** The Spearman correlation coefficient of partial Mantel tests between environmental distance matrices and the dissimilarity matrix of each taxon when the geographic distance was being controlled.

Environmental distance matrix	Vascular plants	Carabidae	Geometridae	Arctiinae
Elevation (Ele)	0.32***	0.26***	0.35***	0.25***
Soil total N (TN)	0.24***	0.03	−0.09	0.11*
Soil texture (ST)	−0.07	−0.03	−0.12	0.19***
Cultivated land (CL)	0.47***	0.28***	0.15**	0.10*
Orchard (OR)	0.08*	−0.14	0.02	−0.07
Woodland (WL)	0.14**	0.02	−0.1	−0.04
Grassland (GL)	0.20***	0.02	−0.02	−0.08
Landscape diversity (SHDI)	0.03	0.04	−0.1	0.01
Semi-natural land area % (SNP)	0.76***	0.23**	0.06*	−0.04

Levels of statistical significance (under one-tailed test of 1000 random permutations): *P < 0.05, **P < 0.01, ***P < 0.001.

**Table 2:** Degree of cross-taxon congruence (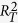, levels of statistical significance (1000 random permutations).

Number of model	Dependent Taxon	Taxon used as Independent variable	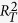	CTG + CTGE	CTE	PT
1	Vascular plants	Carabidae	27.4%***	10.00%	13.00%	4.30%
2	Carabidae	Vascular plants	27.4%***	12.40%	8.00%	7.00%
3	Vascular plants	Geometridae	13.2%***	10.20%	2.80%	0.20%
4	Geometridae	Vascular plants	13.2%***	11.70%	1.30%	0.20%
5	Vascular plants	Arctiinae	4.8%***	4.20%	0.30%	0.30%
6	Arctiinae	Vascular plants	4.8%***	4.30%	0.20%	0.30%
7	Carabidae	Geometridae	21.1%***	18.80%	1.70%	0.60%
8	Geometridae	Carabidae	21.1%***	19.80%	0.90%	0.30%
9	Carabidae	Arctiinae	13.9%***	10.80%	1.90%	1.30%
10	Arctiinae	Carabidae	13.9%***	10.60%	2.10%	1.20%
11	Geometridae	Arctiinae	26.0%***	22.40%	0.40%	3.20%
12	Arctiinae	Geometridae	26.0%***	19.30%	0.90%	5.80%

^***^P < 0.001, representing the congruence of species turnover between two taxa, partitioned into three fractions by variation partitioning, the congruence between two taxa explained by geographic distance (CTG+CTGE), independently by environmental distance (CTE), and PT represents the congruence between two taxa believed to relate to their direct biotic interaction).
