# Supplementary material for: Disentangling effects of abiotic factors and biotic interactions on cross-taxon congruence in species turnover patterns of plants, moths and beetles
Source: Sci Rep. 2016 Apr 1;6:23511. doi: 10.1038/srep23511 (PMC4817036; doi:10.1038/srep23511)
Supplement: Supplementary Information [file srep23511-s1.pdf]

Disentangling effects of abiotic factors and biotic interactions on cross-taxon congruence in species turnover patterns of plants, moths and beetles

Meichun Duan, Yunhui Liu, Zhenrong Yu, Jacques Baudry, Liangtao Li, Changliu Wang, Jan C. Axmacher

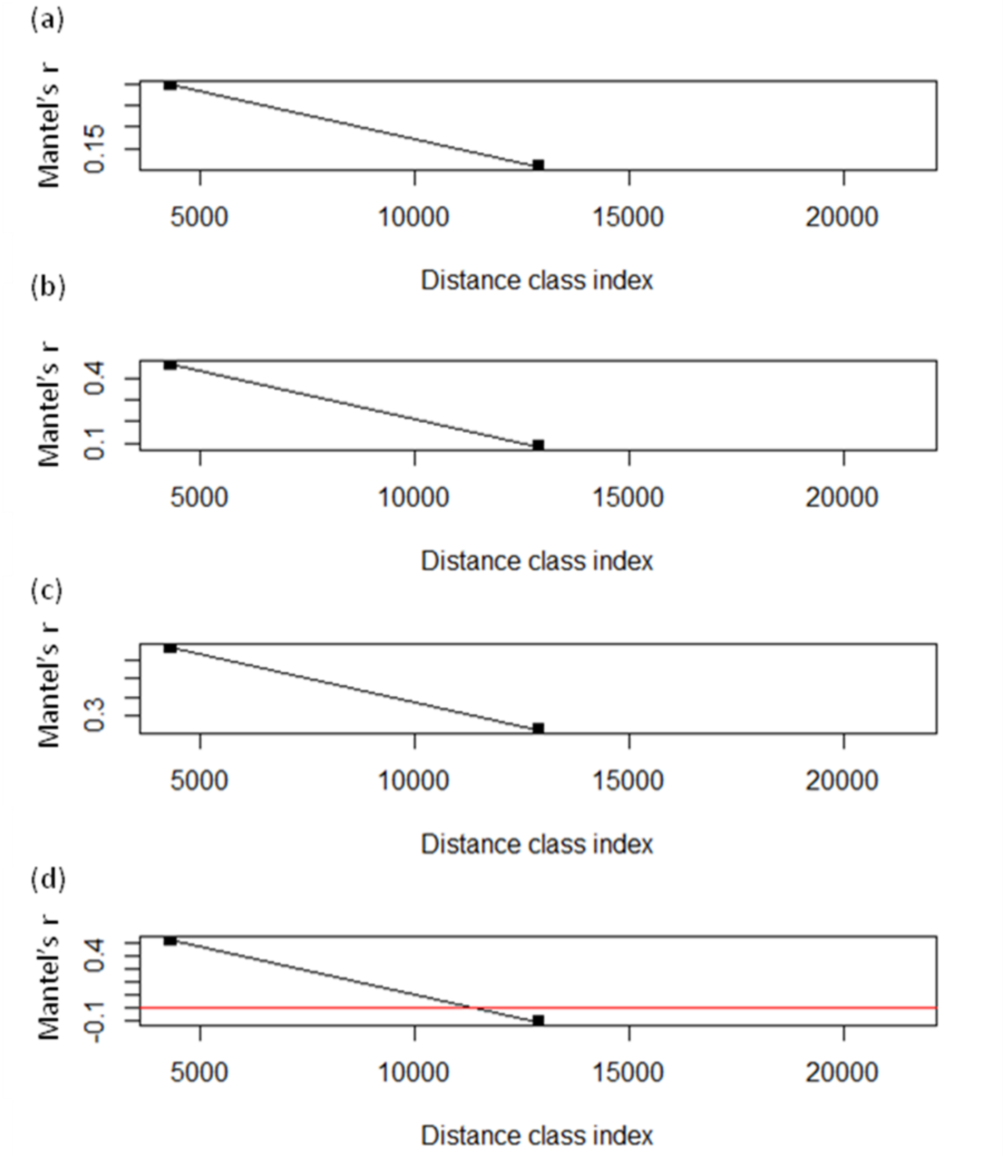

Fig. S1 Mantel correlogram of the Hellinger-transformed species data (a, vascular plant; b, Carabidae; c, Geometridae; d, Arctiinae). Black squares indicate significant multivariate spatial correlation after Holm correction for multiple testing. The abscissa is labeled in meters since this is the unit of the data used to construct the distance classes.

Table S1 Proportions of unique species (species only encountered at this habitat or site) / total species richness of vascular plant, carabid, geometrid and arctiinid assemblages at each habitat and study site.

Study sites	Elevation	Habitat type	Vascular plant	Carabidae	Geometridae	Arctiinae
Dayushu	518-526m	Cultivated land	0.2%/7.2%	1.4%/32.9%	0/27.3%	0/35.0%
		Orchard	0.2%/12.0%	0/15.1%	0/40%	0/45.0%
		Woodland	2.2%/21.2%	0/23.3%	3.6%/50.9%	0/50.0%
		**Total**	**2.9%/28.2%**	**4.1%/43.8%**	**7.3%/59.1%**	**5.0%/55.0%**
Gaojiaying	874-936m	Cultivated land	0.2%/11.3%	1.4%/31.5%	0/30.9%	0/50.0%
		Orchard	4.6%/23.1%	1.4%/37%	0.9%/39.1%	0/40.0%
		Meadow	10.1%/44.8%	2.7%/28.8%	3.6%/55.5%	0/55.0%
		**Total**	**13.7%/60.5%**	**4.1%/60.3%**	**5.5%/60.0%**	**10.0%/70.0%**
Baiqi	1365-1419m	Cultivated land	1.4%/15.7%	6.8%/52.1%	0/40.9%	0/55.0%
		Meadow	1.7%/30.6%	4.1%/38.4%	1.8%/55.5%	0/60.0%
		Woodland	3.1%/36.9%	0/35.6%	7.3%/66.4%	0/60.0%
		**Total**	**10.4%/53.0%**	**12.3%/68.5%**	**15.5%/75.5%**	**0/80.0%**
Shizigou	1621-1679m	Cultivated land	0.5%/16.4%	1.4%/39.7%	0/36.4%	0/50.0%
		Meadow	6.5%/39.8%	4.1%/43.8%	0.9%/41.8%	0/55.0%
		Woodland	5.5%/34.9%	1.4%/26.0%	0/32.7%	0/55.0%
		**Total**	**14.5%/59%**	**8.2%/60.3%**	**2.7%/45.5%**	**0/65.0%**
Total number of species			415	73	110	20
Total number of individuals				3663	14692	1543

Table S2 MRM results based on the selection of different independent variable matrices as the subset of surrogates taxa, with specific resulting R-square terms in each regression representing explanatory variance of dependent taxon. "√" Means this taxon was selected as the independent variable. ***P＜0.001

Dependent Taxon	Independent variable(s)				
	Vascular plants	Carabidae	Geometridae	Arctiinae	R^2
Carabidae	√		√		35.7%***
Arctiinae	√		√		26.1%***
Carabidae	√			√	34.4%***
Geometridae	√			√	32.6%***
Vascular plants		√	√		29.3%***
Arctiinae		√	√		28.4%***
Vascular plants		√		√	27.5%***
Geometridae		√		√	34.4%***
Vascular plants			√	√	13.3%***
Carabidae			√	√	13.9%***

Table S3 Pearson correlation between environmental variables.

	pH	SOM	TN	Ele	ST	CL	OR	WL	GL	SNP	FP
pH	1.00										
SOM	-0.83**	1.00									
TN	-0.76**	0.91**	1.00								
Ele	-0.85**	0.76**	0.69**	1.00							
ST	-0.27	0.19	0.21	0.47**	1.00						
CL	0.03	-0.27	-0.25	-0.03	0.16	1.00					
OR	0.38**	-0.40**	-0.35*	-0.42**	0.04	-.32*	1.00				
WL	-0.34*	0.35*	0.25	0.12	-0.51**	-0.41**	-0.23	1.00			
GL	-0.02	0.29*	0.32*	0.3	0.31*	-0.41**	-0.26	-0.33*	1.00		
SNP	-0.34*	0.6**	0.50**	0.38**	-0.14	-0.67**	-0.42**	0.50**	0.59**	1.00	
FP	0.13	-0.40**	-0.31*	-0.17	0.20	0.81**	0.00	-0.43**	-0.45**	-0.82**	1.00

Levels of statistical significance: *P＜0.05, **P＜0.01

Soil pH (pH); Soil organic matter (SOM); Soil total N (TN); Elevation (Ele); Soil texture (ST); Habitat type: Cultivated land (CL); Habitat type: Orchard (OR); Habitat type: Woodland (WL); Habitat type: Meadow (GL); Shannon-Wiener diversity (SHDI); Semi-natural land % (SNP); Cultivated land % (FP)

Table S4 MRM results based on the selection of different independent variable matrices, with specific resulting R-square terms in each regression representing a unique combination of eight fractions in the variation of taxonomical dissimilarity between plots pairs. PT, pure variation explained by another taxonomical dissimilarity matrix; PE, pure variation explained by environmental distances; PG, pure variation explained by geographic distances; CTE, combined variation explained by another taxonomical dissimilarity and environmental distances; CTG, combined variation explained by another taxonomical dissimilarity and geographic distances; CEG, combined variation explained by environmental and geographic distances; CTEG, combined variation explained by all three matrices and UV, unexplained variance.

Times	Independent variable (s)	R-squares	Combination of fractions
1	Another taxon	R_T^2	PT+CTE+CTG+CTGE
2	Environmental distances	R_E^2	PE+CTE+CEG+CTGE
3	Geographic distances	R_G^2	PG+CTG+CEG+CTGE
4	Another taxon + Environmental distances	R_{TE}^2	PT+PE+CTE+CTG+CEG+CTGE
5	Another taxon+ Geographic distances	R_{TG}^2	PT+PG+CTE+CTG+CEG+CTGE
6	Environmental distances + Geographic distances	R_{EG}^2	PE+PG+CTE+CTG+CEG+CTGE
7	Another taxon + Environmental distances + Geographic distances	R_{TEG}^2	PT+PE+PG+CTE+CTG+CEG+CTGE
